# Prognostic factors of 30-days mortality in primary intracerebral hemorrhage

**DOI:** 10.1186/2197-425X-3-S1-A983

**Published:** 2015-10-01

**Authors:** S Tsikriki, A Karathanou, I Kokoris, T Topalis, E Voutsinas, A Antoniou, E Choli, S Pamouki

**Affiliations:** Intensive Care Unit, General Hospital Volos Ahillopoulio, Volos, Greece

## Introduction

Spontaneous intracerebral hemorrhage(ICH) carries a high mortality rate and predictive factors of short-term outcome are of great importance. Studies have shown that image volumetric evaluation of hematoma, in the initial computed-tomography (CT-scan) of the brain, has an important predictive value of 30-days mortality.

## Objectives

To review patients (pts) with ICH, in a five-year period (January 2010-December 2014) and to assess the predictors of 30-days mortality. As the biggest diameter accounts to the calculation of the hematoma blood volume, we examined a possible association between this diameter and early fatal outcome.

## Methods

Retrospective analysis of 30-days mortality in 62 pts (49 male,13 female, mean age 65,74 ± 11,18) hospitalized in our ICU. Age, Apache II score, Sofa score and GCS were recorded on admission day. CT-scan was performed in order to determine the site of the ICH, the presence of intraventricular hemorrhage (IVH) and the amount of ICH. Volume was estimated using the ABC/2 method and the biggest diameter was recorded separately. Statistical analysis was performed by using SPSS V-20 soft-ware. Variables were described using mean and SD (continues variables) or category percentages (categorical variables), stratified for survivors and nonsurvivors. Independent Samples t-test for Equality of Means, Shapiro-Wilk test of Normality, Pearson correlation and Pearson Chi-Square test were used and level of significance was set at p < 0,05.

## Results

38 pts (group I) nonsurvived in the ICU during the first month and 24 pts (group II) survived and discharged from acute hospitalization.The overall mortality was 61,29%.See results in table 1.

**Table 1:** Table 1

	Group I	Group II	p value
Age	65,97 ± 11,57	65,38 ± 10,76	p = 0,8
Apache II score	23,13 ± 5,60	15,63 ± 6,14	p < 0,0001
Sofa score	9,16 ± 2,66	5,96 ± 2,56	p < 0,0001
GCS	4,76 ± 1,79	8,79 ± 3,09	p < 0,0001
ICH volume (cm3)	62,68 ± 23,20	27,42 ± 23,22	p < 0,0001
biggest diameter (BD) (cm)	6,9 ± 1,26	4,58 ± 1,60	p < 0,0001

*[Comparison of 30-days mortality]*

Biggest diameter of hematoma and ICH volume have a positive linear correlation (Pearson correlation R^2^ = 0,664). Furthermore, we attempted to establish a possible association between the BD and the 30-days mortality. We divided our pts in: Group A (12 pts):BD ≤ 4 cm and Group B ( 50pts): BD > 4cm. One patient died in group A (8,3%), while 37 pts died in group B (74%) (Pearson Chi-square test p < 0,0001,odds ratio:0,032).

## Conclusions

We demonstrated that Apache II score,Sofa score, GCS on admittion and ICH volume are independent predictive factors of 30-days mortality in pts with ICH. The size of the biggest diameter (cut off 4 cm) has a strong association with the 30-days mortality, thereby it could be an independent predictive factor in these group of patients.
